# The emerging possibility of the use of geniposide in the treatment of cerebral diseases: a review

**DOI:** 10.1186/s13020-021-00486-3

**Published:** 2021-08-28

**Authors:** Wenwen Zhang, Fangling Zhang, Qichao Hu, Xiaolin Xiao, Linbo Ou, Yuan Chen, Shiqing Luo, Yonghong Cheng, Yinxiao Jiang, Xiao Ma, Yanling Zhao

**Affiliations:** 1 State Key Laboratory of Southwestern Chinese Medicine Resources, School of Pharmacy, Chengdu University of Traditional Chinese Medicine, Chengdu, 611137 China; 2 Hospital of Chengdu University of Traditional Chinese Medicine, School of Clinical Medicine, Chengdu University of Traditional Chinese Medicine, Chengdu, 611137 China; 3 College of Health and Rehabilitation, Chengdu University of Traditional Chinese Medicine, Chengdu, 611137 China; 4 Department of Pharmacy, The Fifth Medical Centre of PLA General Hospital, Beijing, 100039 China

**Keywords:** Geniposide, Cerebral ischaemia, Alzheimer’s disease, Depression, Mechanism

## Abstract

With the advanced discoveries in the field of pathogenesis, a series of cerebral diseases, such as cerebral ischaemia, Alzheimer's disease, and depression, have been found to have multiple signalling targets in the microenvironment. Only a few existing agents have been shown to have curative effects due to this specific circumstance. In recent decades, active ingredients isolated from natural plants have been shown to be crucial for original drug development. Geniposide, mainly extracted from *Gardenia jasminoides* Ellis, is representative of these natural products. Geniposide demonstrates various biological activities in the treatment of cerebral, cardiovascular, hepatic, tumorous, and other diseases. The multiple protective effects of geniposide on the brain have especially drawn increasing attention. Thus, this article specifically reviews the characteristics of current models of cerebral ischaemia and illustrates the possible effects of geniposide and its pathogenetic mechanisms on these models. Geniposide has been shown to significantly reduce the area of cerebral infarction and alleviate neuronal damage and necrosis mainly by inhibiting inflammatory signals, including NLRP3, TNF-α, IL-6, and IL-1β. Neuronal protection was also involved in activating the PI3K/Akt and Wnt/catenin pathways. Geniposide was able to increase autophagy and inhibit apoptosis by regulating the function of mTOR in treating Alzheimer's disease. Geniposide has also been shown to act as a glucagon-like peptide-1 receptor (GLP-1R) agonist to reduce amyloid plaques and inhibit oxidative stress to alleviate memory impairment as well as synaptic loss. Moreover, geniposide has been shown to exert antidepressant effects primarily by regulating the hypothalamic–pituitary–adrenal (HPA) axis. Detailed explorations have shown that the biological activities of inhibiting inflammatory cytokine secretion, alleviating oxidative stress, and suppressing mitochondrial damage are also involved in the mechanism of action of geniposide. Therefore, geniposide is a promising agent awaiting further exploration for the treatment of cerebral diseases via various phenotypes or signalling pathways.

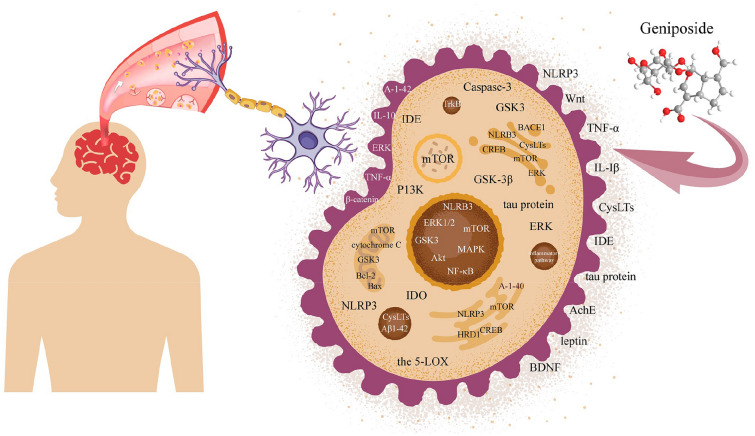

## Introduction

**Fig. 1:**
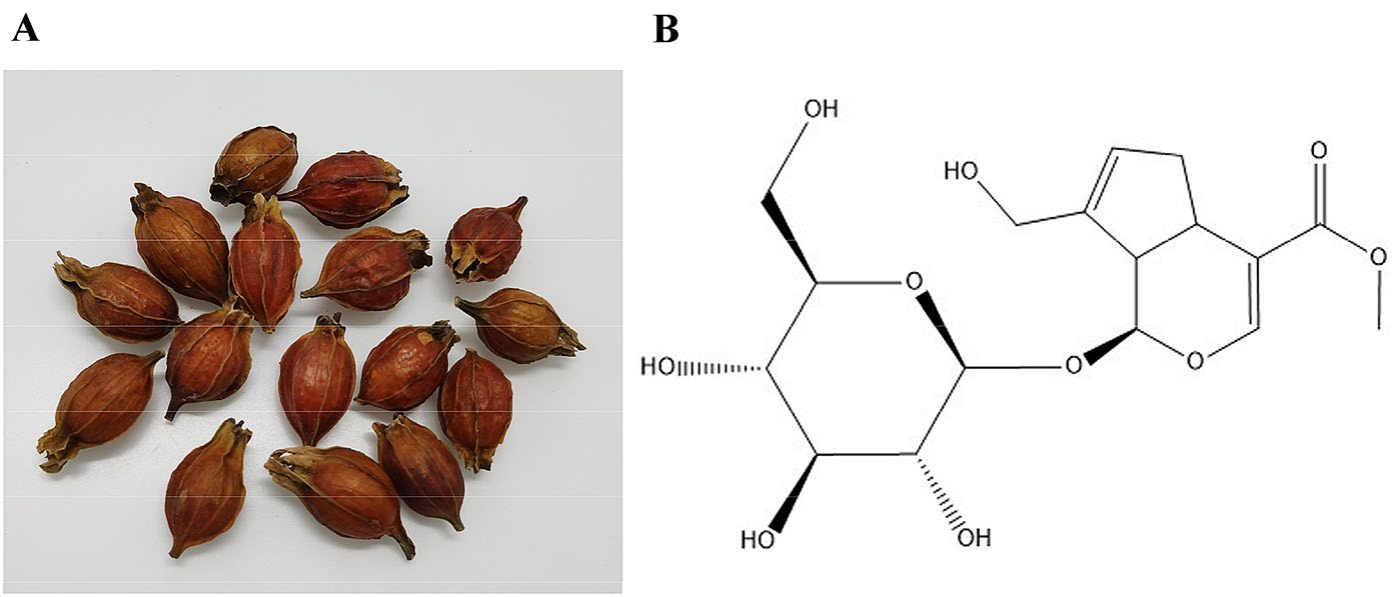
**A** The plant *Gardenia jasminoides* Ellis. **B** Molecular structure of geniposide

Natural products, mainly agents from plants, have been proven to have wide pharmacological activities over the decades. It is believed that the active ingredients of natural products can be used for many complex diseases according to the developed recognition of pathogenesis. Thus, research on natural products in various diseases has vastly increased in recent years [1, 2]. As one of the representative iridoid glucosides, geniposide has received much attention for its traditional applications and in modern pharmacological studies. Geniposide is extracted from the fruit of Rubiaceae *Gardenia jasminoides* Ellis (Fig. 1). Geniposide has a long history of clinical application according to the record of *Shen Nong's Materia Medic*a. It is worth mentioning that *Gardenia jasminoides* Ellis is also among the first batch of medicine and food resources issued by the Ministry of Health of China [3–5]. As a traditional Chinese herbal medicine with important development potential, various studies in recent decades have confirmed that gardenia has a wide range of pharmacological activities. Geniposide is effective in antipyretic, sedative, analgesic, anti-inflammation, antipathogenic, antioxidative, and antiradiation treatments. Geniposide also demonstrates significant antitumour biological activities, lowering blood sugar, lowering blood fat, protecting the liver and gall bladder, protecting the gastric mucosa, and improving depression [6–8]. Hitherto, geniposide has been widely used for liver and gallbladder disorders in the clinic. Its modern application is extended to antiviral treatment, and geniposide also provides cerebral protection with robust efficacy. A series of studies revealed the biological activities of geniposide in downregulating phenotypes, including oxidative stress, inflammation, and apoptosis. Other kinds of functions, such as immune regulation and neuroprotection, have also been reported in various diseases [9–11]. Apart from a single application, the combination of geniposide with baicalin, ginsenoside, and chlorogenic acid for complex diseases has also drawn much focus [12, 13]. Therefore, it is crucial for current researchers to systematically sort out the relationship between the pharmacologic effects and mechanisms of geniposide.

In recent years, the high incidence of cerebral diseases has aroused attention due to the associated low quality of life, high disability rate, and high fatality rate worldwide [14, 15]. On the other hand, cerebral disease are more difficult to treat than other kinds of organic diseases. Only a few low-molecular-weight compounds can reach the brain tissue to exert curative effects due to the existence of the blood–brain barrier (BBB) [16, 17]. Therefore, cerebral diseases are bound to cause a heavy burden on current society. Cerebral ischaemia, Alzheimer's disease, and depression are thought to be representative brain diseases. Cerebral ischaemia, accounting for 7/10 incidences of stroke, which is the third most common cause of death, with an incidence rate of 150–21,740/100,000 people per year, is characterized by a high disability rate, high recurrence rate, and high fatality rate [18, 19]. Unfortunately, the current main therapies, including antiplatelet and thrombolytic agents, are thought to have great limitations. The main therapies are often accompanied by potential cerebral haemorrhage risks and with narrow treatment times. Thus, the application is often not available in time, resulting in the aggravation of the disease and higher mortality, especially in developing areas [20–24]. The treatment of Alzheimer's disease also faces a similar severe situation. Dementia is a group of clinical syndromes referring to chronic acquired progressive mental deterioration. Dementia is mainly characterized by slow-onset intellectual decline, accompanied by varying degrees of personality changes. Alzheimer's disease is the main cause of dementia, accounting for 80% of dementia cases. With the development of an ageing society, Alzheimer's disease is considered to be the world's major public health problem and has been identified as a priority research project. However, due to the complex pathogenesis of Alzheimer’s disease, the current clinical treatment can relieve symptoms and improve the quality of life to a certain degree but it cannot postpone the progression of nor reverse the disease [25, 26]. Depression is a central psychiatric disease with a vastly increasing number of patients in recent years. Depression clinically manifests as abnormal mood and behaviour, often accompanied by a strong suicidal tendency and autonomic or somatic symptoms. According to the World Health Organization statistics, 320 million people suffer from depression worldwide, and depression is expected to become the world's second most common disease. The annual death toll from suicide due to depression has reached 1 million. Depression may lead to psychosocial disorders, decreased quality of life, and high morbidity, accompanied by high disability and high mortality. Although various antidepressants are used to treat depression, antidepressants show poor performance in at least 50% of patients. Patient response to long-term antidepressant treatment is far from satisfactory [27–29] (Table 1)

**Table 1:** Research progress on geniposide in the treatment of brain diseases

Disease	Animal/Cell model	Dosage	Target/Pathways/Mechanism	References
Cerebral ischaemia	tMCAO	25–150 mg/kg	GluN2A/AKT/ERK signalling pathways (+)	[37]
	Permanent bilateral common carotid arteries occlusions	50–100 mg/kg	NF-κB/iNOS/TNF-α/IL-6 (−)	[38]
	MCAO	15–60 mg/kg	inflammatory factors and NF-κB (−)	[39]
	I/R	100 mg/kg	AKT/mTOR signalling pathways (+)	[45]
	OGD/R	6–12 μg/mL	NLRP3 (−)	[49]
	OGD	12.5–50 μg/mL	inflammatory factors/NF-κB (−)	[39]
	OGD	100–300 μM	PI3K/AKT and Wnt/β-catenin pathways (+)	[53]
	OGD	33.2 μg/mL	ERK1/2 signalling pathways/proinflammatory cytokines (−)	[54]
	Inflammatory injury induced by microglia	12.5–50 µg/mL	5-LOX/CysLTs inflammatory pathway (−)	[55]
	OGD	31.25–500 µg/mL	CHOP/Beclin 1 (−)	[56]
Alzheimer's disease	APP/PS1	50 mg/kg	CcO (+)ROS/MDA (−)	[63]
	APP/PS1	50 mg/kg	mTOR signalling pathway (−)	[64]
	APP/PS1	5–20 mg/kg	Akt/GSK-3β/tau (+)	[65]
	APP/PS1	12.5–50 mg/kg	MAPK signalling pathway (−)	[66]
	APP/PS1	12.5–50 mg/kg	cytochrome c oxidase (+)	[67]
	STZ	5–20 mg/kg	BACE1/IDE (+)ADAM10 (−)	[68]
	STZ	50 μM	GSK3β (−)	[69]
	STZ	12.5–25 mg/kg	Aβ1-42 (−)	[70]
	Primary cultured cortical neurons	10 µM	Akt/GSK-3β/tau (+)	[65]
	Primary cultured cortical neurons	10 µM	ADAM10/A-1-42 (−)	[68]
	Primary cultured cortical neurons	2.5–10 µM	AChE (−)	[66]
	Primary cultured cortical neurons	10 µM	HRD1 (+)	[71]
	Primary cultured cortical neurons	0.01–20 µM	leptin (+)	[72]
	Primary cultured cortical neurons	10 µM	JAK2/STAT3 (−)	[73]
	PC12 cells	50 µmol/L	PI3K (+)	[81]
	PC12 cells	25–100 mg/L	p90RSK (+)	[82]
Depression	RRS	50–100 mg/kg	GLP-1R/AKT (+)	[86]
	CUMS	25–100 mg/kg	HPA axis (−)	[87]
	STZ	50–100 mg/kg	TrkB/BDNF (+)	[88]
	HDF in combined with CORT	100 mg/kg	CREB (+)	[89]
	Lipopolysaccharide	100 mg/kg	NF-κB/IDO (−)	[90]
Others	Maximal electric shock (50 mA, 50 Hz, 1 s)	5–20 mg/kg	PI3K/Akt/GSK-3β (+)	[91]
	1-Methyl-4-phenyl-1,2,3,6-tetrahydropyridine	100 mg/kg	Caspase 3/Bcl-2 (−)Bax (+)	[92]
	1-Methyl-4-phenyl-1,2,3,6-tetrahydropyridine/neuroblastoma cell lines SHSY5Y	100 mg/kg	LAMP2A/α-synuclein(+)	[93]

tMCAO: transient middle cerebral artery occlusion; MCAO: middle cerebral artery occlusion; I/R: ischaemia/reperfusion; OGD: oxygen–glucose deprivation; STZ: streptozotocin; RRS: repeated restraint stress; CUMS: chronic unpredictable mild stress; CORT: corticosterone

Many studies have focused on the therapeutic effect of geniposide. There is currently a lack of comprehensive reviews. To obtain close insight into the mechanism by which geniposide treats cerebral diseases, this article first introduces multiple recognized models for agent exploration. Then, this article reviews the current status of research on geniposide for the treatment of cerebral ischaemia, Alzheimer's disease (AD) and depression. Furthermore, the signals and targets of geniposide for brain protection are specifically summarized.

## Cerebral ischaemia

### Main research models

The current study found that research on geniposide treatment of cerebral ischaemia is mainly related to three pathogenic models: middle cerebral artery occlusion (MCAO), cerebral ischaemia/reperfusion (I/R) and oxygen–glucose deprivation (OGD). Among them, MCAO is the most common model. The middle cerebral artery (MCA) and its branches are the cerebral blood vessels most affected by cerebral ischaemia in humans. Ischaemic stroke caused by vascular occlusion accounts for 85% of all causes of cerebral ischaemia. Therefore, surgical suture occlusion of arteries to block the blood supply from the front and rear arteries is the most commonly used method to simulate human cerebral ischaemic diseases and has been widely used in rodents. The MCAO model is less traumatic and does not require complicated craniotomy, thus avoiding damage to the brain structure [30–32]. After long-lasting cerebral ischaemia, tissue plasminogen activator (tPA) thrombolytic therapy is often used to restore the blood supply to brain tissue. Although timely recanalization of blood vessels after cerebral ischaemia is the most direct and effective treatment method, the core of brain tissue dies within a few minutes after the onset of cerebral ischaemia, causing irreversible damage. Reperfusion itself can cause additional damage to the ischaemic penumbra, which is the area adjacent to the core of the infarction, leading to so-called I/R injury [33, 34]. This is precisely because reperfusion causes new damage; therefore, this model can be used to develop neuroprotective drugs. This model causes a variety of damage to the cerebral microcirculation, so it is also widely used to simulate ischaemic injury in stroke patients. Compared with MCAO, ischaemia–reperfusion can be used to deliver drugs to the infarct area and maximize the therapeutic effect [35–37]. On the other hand, ischaemia also represents an interruption of metabolic energy and oxygen delivery, therefore cell oxidative metabolism cannot be supported. The reintroduction of oxygen during the reperfusion of ischaemic tissue triggers oxidative stress, which triggers a reperfusion injury cascade and ultimately leads to cell and tissue damage and death. Therefore, the OGD model is to culture cells under hypoxia, energy deprivation and reoxygenation conditions to simulate the pathological characteristics of ischaemia–reperfusion in vitro. At present, three-gas incubators are often used internationally to cause hypoxia within the cells under the conditions of low oxygen and high nitrogen, along with low-sugar and serum-free medium to cultivate the cells. However, the main disadvantage of this model is the high requirements for equipment [38–40].

### The potential mechanisms of geniposide in MCAO models

The transient middle cerebral artery occlusion (tMCAO) model is one of the main models in the current MCAO model, which is a transient cerebral ischaemia model. Because the recanalization of blood flow in human stroke is slow and gradual, this model is closer to human stroke than other models. However, this model is extremely difficult to operate, so the permanent middle cerebral artery occlusion (pMCAO) model is more widely used in animal models. Studies have reported that geniposide could significantly reduce the area of cerebral infarction when 75 mg/kg was injected intraperitoneally to treat MCAO model rats, while improving antiapoptotic functions and inhibiting blood–brain barrier (BBB) leakage/haemorrhage by elevating GluN2A-containing *N*-methyl-d-aspartate receptor (NMDAR) expression in tMCAO rats. In addition, the protective effect of geniposide was also due to the enhancement of GluN2A-dependent survival signals, including pAKT, pERK and PSD-95. However, the overall effect of geniposide on the treatment of cerebral ischaemia in the MCAO rat model was dose-dependent, and there was no significant neuroprotective effect at the low dose of 25 mg/kg and the high dose of 150 mg/kg [41]. Other studies in a rat MCAO cerebral ischaemia model showed that both 50 mg/kg and 100 mg/kg doses could significantly improve the loss of nerve cells, morphological damage, and nuclear rupture. At the same time, geniposide could reduce inducible nitric oxide synthase (iNOS) and nuclear factor-kappa B (NF-κB) expression and inhibit the release of inflammatory factors tumour necrosis factor-α (TNF-α) and interleukin-6 (IL-6) to reduce neuroinflammation. In addition, the study also proved that geniposide could also effectively prevent cognitive decline in rats due to chronic cerebral hypoperfusion [42]. In a study, rats were given high-dose (60 mg/kg), medium-dose (30 mg/kg), and low-dose (15 mg/kg) geniposide immediately after MCAO. The results showed that the middle-dose and high-dose groups significantly had reduced cerebral infarction rates by 32.9% and 47%, respectively [43]. It is worth mentioning that Huanglian Jiedu Decoction, which uses geniposide as the main compatibility drug, has been extensively researched in the treatment of neurodegenerative diseases. Huanglian Jiedu Decoction is mainly composed of Rhizoma Coptidis (*Coptis chinensis* Franc., Ranunculaceae), Radix Scutellariae (*Scutellaria baicalensis* George., Lamiaceae), Cortex Phellodendri (*Phellodendron amurense* Rupr., Rutaceae), and Fructus Gardeniae (*Gardenia jasminoides* Ellis., Rubiaceae) [44, 45]. Therefore, there have also been many studies investigating the effect of geniposide on cerebral ischaemia and its compatibility in Huanglian Jiedu Decoction. Studies have found that the pharmacokinetics of geniposide correspond to its antioxidant effect, and it plays a role in all aspects of antioxidant biological regulation [46]. In addition, studies have also found that geniposide has a great effect in treating cerebral ischaemia by improving metabolic abnormalities, regulating oxidative stress, inhibiting neuronal apoptosis, and reducing inflammation. It has also been found that combining geniposide with drugs such as berberine and baicalin and other monomers has the potential to enhance treatment efficacy [47, 48].

The BBB is involved in the pathogenesis of ischaemic stroke. Therefore, we found that geniposide can also be used to restore BBB function. Some studies have established an in vitro BBB model composed of primary cultures of brain microvascular endothelial cells and astrocytes to study the effect of geniposide on the BBB. The results showed that geniposide pretreatment could reduce the permeability of the BBB, promote the expression of tight junction proteins (zonula occludens-1, claudin-5 and occludin), increase transendothelial resistance, and effectively improve blood–brain barrier function. In addition, geniposide could reduce oxidative stress damage and release inflammatory factors, downregulate the expression levels of matrix metallopeptidase-9 (MMP-9) and MMP-2, and increase brain-derived neurotrophic factor and glial cell-derived release of neurotrophic factors. Geniposide was shown to restore BBB function through the above effects [49].

### The potential mechanisms of geniposide in cerebral I/R models

Some studies have established an I/R model and administered 100 mg/kg geniposide. The results showed that geniposide could reduce the area of myocardial infarction, reduce acute myocardial injury, improve heart function, regulate apoptosis-related proteins, and inhibit cell apoptosis. In addition, geniposide could inhibit the expression of autophagy-related proteins and the accumulation of autophagosomes in vivo and in vitro. Through the above effects, geniposide was shown to improve I/R damage by activating the AKT/mTOR signalling pathway [50].

### The potential mechanisms of geniposide in in vitro OGD models

**Fig. 2:**
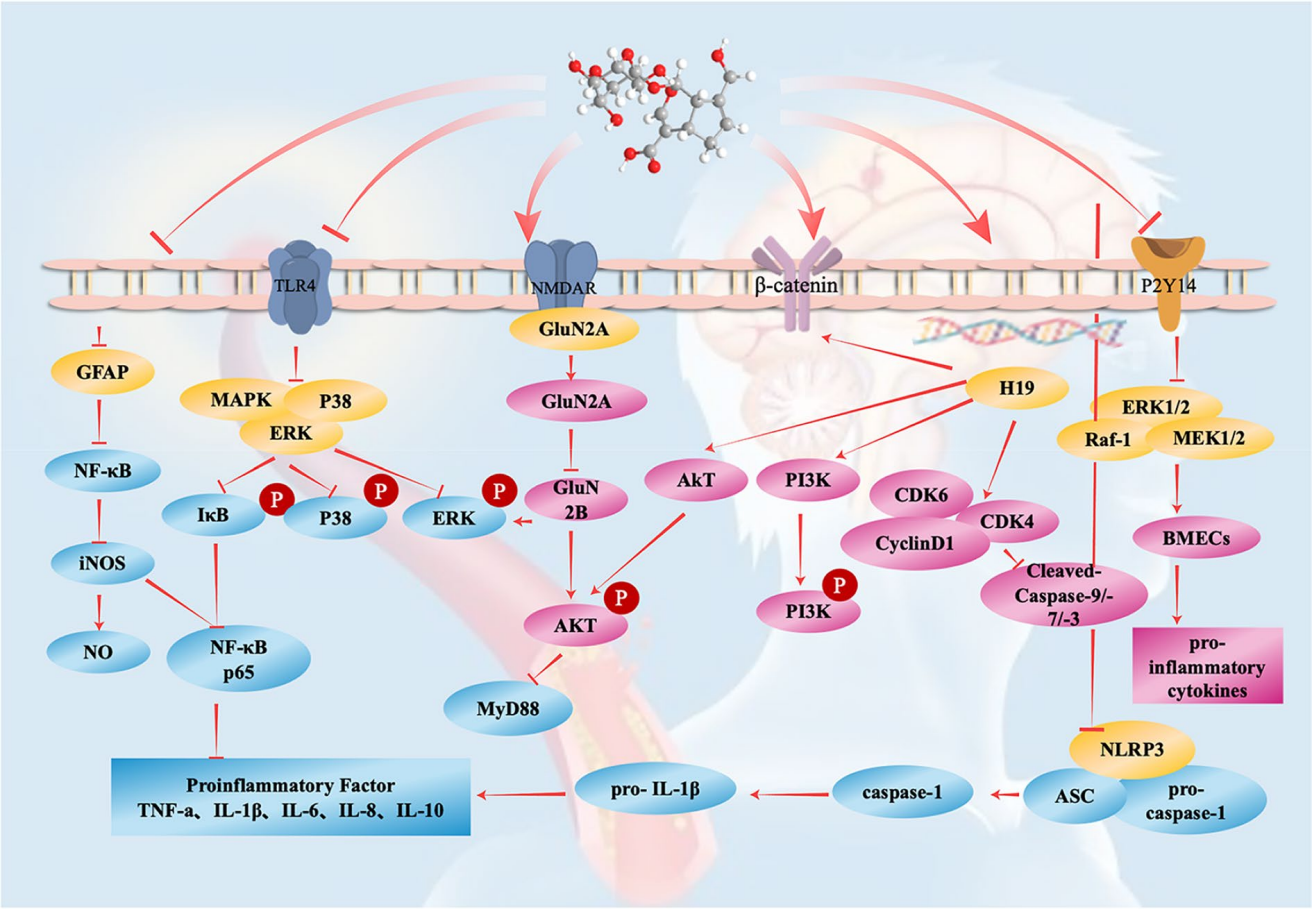
The therapeutic effect of geniposide on cerebral ischaemic disease. Geniposide enhances GluN2A-dependent survival signals, including pAKT, pERK and PSD-95, and works by reducing the expression of iNOS and NF-κB and inhibiting the release of the inflammatory factors tumour necrosis factor-α (TNF-α) and IL-6. In addition, geniposide activates the PI3K/AKT and Wnt/catenin pathways by upregulating the expression of H19 in PC-12 cells

Microglia are inherent immune effector cells in the central nervous system. They mediate the endogenous immune response of central nervous system injuries and diseases and play an extremely important role in the physiological processes of the central nervous system. Microglia are involved in a series of neurodegenerative diseases. Microglial activation and neuroinflammation are the main features of neuropathology [51–53]. Therefore, many studies have evaluated the specific mechanism of geniposide in the treatment of cerebral ischaemic diseases in a microglial OGD model. Studies have shown that geniposide can inhibit the expression of the nod-like receptor protein 3 (NLRP3) inflammasome and inflammatory cytokines, thereby activating the autophagy activity of microglia, reducing inflammation, and protecting neurons. In this study, geniposide mainly reduced the expression of NLRP3, ASC (CARD domain), cleaved caspase-1, interleukin-1 (IL-1) and P62 and increased the conversion of LC3 and Beclin-1 [54]. Another study found that OGD could activate microglia and promote tumour necrosis factor-α (TNF-α), interleukin-1β (IL-1β), interleukin-6 (IL-6), interleukin-8 (IL-8) and interleukin-10 (IL-10), but the abovementioned effects of OGD in rat primary microglia after treatment with geniposide were inhibited, and geniposide could also inhibit OGD-induced Toll-like receptor 4 (TLR4) transcription and translation. It is worth noting that the phosphorylation levels of ERK, IκB and p38 in microglia treated with 25 and 50 μg/mL geniposide were significantly reduced, and geniposide at these two concentrations could inhibit NF-κB p65-triggered nuclear transcription activity, thereby inhibiting the activation of microglia [39]. The PC-12 cell line is derived from pheochromocytoma in the adrenal medulla of adult rats. The generalized PC-12 cell line includes the cells before differentiation into nerve cells and glial cells and all the nerve cells and glial cells formed by their evolution [55–57]. Geniposide also has a protective effect on PC-12 cells in an OGD cerebral ischaemia model. First, geniposide was shown to significantly reduce the damage to PC-12 cells in an OGD model. Second, geniposide was shown to activate the PI3K/Akt and Wnt/catenin pathways by upregulating the expression of H19 in PC-12 cells in an OGD model, thus playing a protective role in cells [58]. In addition, there have also been studies on the therapeutic mechanism of geniposide in cerebral ischaemia by establishing brain microvascular endothelial cell (BMEC) OGD in vitro cerebral ischaemia models. Studies have shown that the transcription and translation of P2Y14 receptors in BMECs of rats with ischaemia–reperfusion injury increase. Geniposide can significantly inhibit the phosphorylation of RAF-1, mitogen-activated protein kinase 1/2 (MEK1/2), and extracellular signal-regulated kinase 1/2 (ERK1/2) to reduce the expression of the P2Y14 receptor and inhibit the release of the proinflammatory cytokines IL-8, IL-1 and monocyte chemotactic protein 1 (MCP-1) in BMECs induced by OGD [59]. Similarly, there are related literature reports on the therapeutic effect of baicalin and geniposide on a microglial inflammatory injury model established by lipopolysaccharide (LPS) stimulation of BV-2 cells in vitro. The literature shows that the combination of baicalin and geniposide can adjust the polarization state of microglia, downregulate 5-LOX/Cylts, and play a protective role against inflammation and nerve damage [60]. Studies have also found that the pretreatment of cells with geniposide can increase cell viability. In addition, the combined use of geniposide and tauroursodeoxycholic acid (TUDCA) was shown to have a stronger therapeutic effect on damaged cells than treatment with geniposide alone. Further results showed that the protective effect of geniposide and TUDCA on OGD/R model cells was related to endoplasmic reticulum stress and the autophagy of related cells [61] (Fig. 2).

## Alzheimer's disease

### Main research models

At present, the main models of AD research are the APP/PS1 transgenic mouse model and the AD model induced by streptozotocin (STZ). The deletion of the ninth exon in DeltaE9 of the human presenilin gene can cause a DeltaE9 mutation, which can cause early-onset AD. The APP/PS1 double transgenic mice can express a mutant human presenilin (DeltaE9) and human-mouse amyloid preprotein (APPswe) fusion. Therefore, the use of APP/PS1 double transgenic mice has unique genetic advantages, and these mice have become an ideal model for studying the pathogenesis of AD [62–64]. Streptozotocin belongs to the family of glucosamine-nitrosourea compounds, which are DNA alkylation reagents that can enter cells alone through GLUT2 (glucose transport protein). Streptozotocin is toxic to β-cells induced by pancreatic islet insulin, thereby inducing an insulin deficiency state, which may be the basis of the neuropathogenesis of sporadic AD [65–67].

### The potential mechanisms of geniposide in APP/PS1 double transgenic mice

The literature has reported that geniposide can regulate the activity of the mechanistic target of rapamycin by activating glucagon-like peptide-1 (GLP-1) receptors. At the same time, geniposide can also increase autophagy and inhibit cell apoptosis by regulating the function of mTOR, thereby improving the neuropathological damage of AD. The results show that treatment of APP/PS1 mice with geniposide can not only improve cognitive deficits but also reduce amyloid-β 1-40 (Aβ1-40) plaque deposition and reduce soluble Aβ1-40 and Aβ1-42 in APP/PS1 mice. The expression of p-Akt/Akt and p-mTOR/mTOR was reduced, and the expression of p-4E-BP1/4E-BP1, LC3-II and Beclin1 was increased. All these results indicate that geniposide can enhance autophagy and lysosome clearance of Aβ fibres by downregulating mTOR signalling, which is the basis for geniposide to treat AD and exert its protective effect [68].

Another study showed that gavage of APP/PS1 mice with geniposide at 50 mg/kg/d can effectively improve mice's exploration and memory abilities and effectively improve cognitive impairment. Geniposide exerts a neuroprotective effect by increasing the expression level of p-4E-BP1 and reducing the expression of p-mTOR and p-Akt, thereby improving cognitive impairment. This effect is mediated through the regulation of mTOR-related protein pathways [69]. In addition, some studies have found that geniposide treatment for 4 weeks could significantly reduce the phosphorylation level of tau protein in the brains of APP/PS1 transgenic mice and accelerate the phosphorylation of glycogen synthase kinase-3 (GSK3) [70]. Another study found that geniposide could inhibit the excessive activation of MAPK signals mediated by rage interaction and improve the accumulation of cholinergic defects in the brain's hippocampus, thereby improving learning and memory impairment [71]. Oxidative stress and mitochondrial dysfunction appear in the early stage of AD and accelerate the development of the disease. Studies have pointed out that the gavage of APP/PS1 mice with geniposide could inhibit mitochondrial oxidative damage, increase mitochondrial membrane potential (MMP) and cytochrome c oxidase (CcO) activity, and prevent the development of AD by significantly improving oxidative stress and mitochondrial dysfunction in mice [72].

### The potential mechanisms of geniposide in streptozotocin-induced Alzheimer's disease models

Studies have shown that 90 mg/kg STZ administered to wild-type and APP/PS1 transgenic mice daily for 2 consecutive days could significantly reduce the mice's peripheral and brain insulin levels. However, continuous treatment with geniposide for 4 weeks not only significantly reduced the concentration of β-amyloid peptides (Aβ1-40 and Aβ1-42) in the brains of AD mice induced by STZ but also increased the protein levels of β-site APP cleaving enzyme (BACE1) and insulin-degrading enzyme (IDE) and reduced the protein level of ADAM10. The authors of the study believe that the findings effectively confirm the therapeutic link between diabetes and AD and may confirm that geniposide will become a new drug for the treatment of AD [73]. Another study found that geniposide could improve the symptoms of type 2 diabetes, and a single injection of geniposide (50 μM, 10 μL) into the lateral ventricle of STZ-induced AD rats could effectively reduce approximately 40% of spatial learning defects, downregulating the level of tau phosphorylation by approximately 30%. The study found that STZ could increase the activity of GSK3β and that tau protein could be phosphorylated by GSK3. Geniposide reduces the activity of GSK3β induced by STZ by increasing the expression level of GSK3β (pS-9) and inhibiting the expression level of GSK3β (pY-216). In addition, a study found through ultrastructural analysis that geniposide did not participate in STZ-induced neuropathy, such as helical fibre (PHF)-like structure, the accumulation of vesicles at the end of the synapse, abnormal endoplasmic reticulum (ER) and apoptosis [74]. Early in the study, it was discovered that geniposide not only stimulated GLP-1R but also regulated the secretion of glucose-stimulated insulin in vitro, which has nutritional and neuroprotective effects. Studies have found that while geniposide regulates insulin and blood sugar levels, it also reduces the level of A-g1-42 and upregulates the expression of insulin-degrading enzymes. The enzyme plays an irreplaceable role in the degradation of a polypeptide. These research results will be combined with those from the study of neuronal dysfunction in diabetes to find new methods for the treatment of AD [75].

### The potential mechanisms of geniposide in primary cultured cortical neurons

The study of the therapeutic effect of geniposide on AD by culturing primary cultured cortical neurons in vitro is one of the most commonly used methods in current research. Studies have found that in primary cultured cortical neurons, geniposide could directly upregulate the phosphorylation level of Akt, downregulate the phosphorylation level of tau protein, and enhance the phosphorylation of tau protein and Akt and GSK-3 cells in cortical neurons. This effect was inhibited by preincubation with the PI3K inhibitor LY294002. It can be seen from this study that geniposide may play a key role in the treatment of AD by enhancing insulin signalling and the phosphorylation of tau protein [70]. Another study also found that geniposide can directly enhance the effect of insulin by reducing the level of A-1-42 in primary cultured cortical neurons [73]. There is also a report about A-1-42 in another literature. The literature shows that geniposide reduces the activity of acetyl-cholinesterase (AChE) by downregulating the choline acetyltransferase (ChAT) level and the activity of cultured primary hippocampal neurons, thereby inhibiting oligomers. The toxic effect of A-2-42 induced cholinergic deficiency. These results indicate that geniposide can enhance memory by enhancing cholinergic neurotransmission [71]. Studies have found that the activation of the unfolded protein response (UPR) is a signalling pathway that enhances adaptive procedures to maintain protein stability. The changes in protein stability caused by tau protein hyperphosphorylation and amyloid peptide aggregation are precise, characteristic changes in AD. Through high glucose administration to induce significant activation of UPR in primary cortical neurons, and then treatment with geniposide, we found that geniposide not only enhanced the phosphorylation of IRE1 (the most conservative UPR signalling branch) by high glucose but also upregulated the expression level of HRD1 (ubiquitin ligase E3) induced by high glucose in primary cortical neurons and made the expression of HRD1 time-dependent. However, the use of STF-083010, an inhibitor of IRE1 phosphorylation, can inhibit the abovementioned effects of geniposide and inhibit the cellular activity of IRE1. It is worth mentioning that HRD1 is also involved in geniposide-mediated regulation of APP degradation in primary cortical neurons. The analysis of these data shows that geniposide can restore protein stability by activating the UPR, thereby playing a role in the treatment of AD [76]. Another study on the leptin receptor signalling pathway showed that geniposide not only significantly enhanced the expression of leptin receptors and reduced the phosphorylation level of tau protein but also enhanced Akt phosphorylation at Ser473 and GSK-3 phosphorylation at Ser-9. However, these effects of geniposide can be inhibited by leptin antagonist (LA). This study shows that geniposide can treat AD by regulating the leptin receptor signalling pathway [77]. In another study, primary neurons were first treated with LA and then geniposide. The study found that geniposide could induce the phosphorylation of JAK2 and STAT3 and regulate the expression levels of α- and β-secretase. The results indicate that geniposide may regulate the production of Aβ1-42 through leptin signalling [78].

### GLP-1R is a potential target of geniposide

**Fig. 3:**
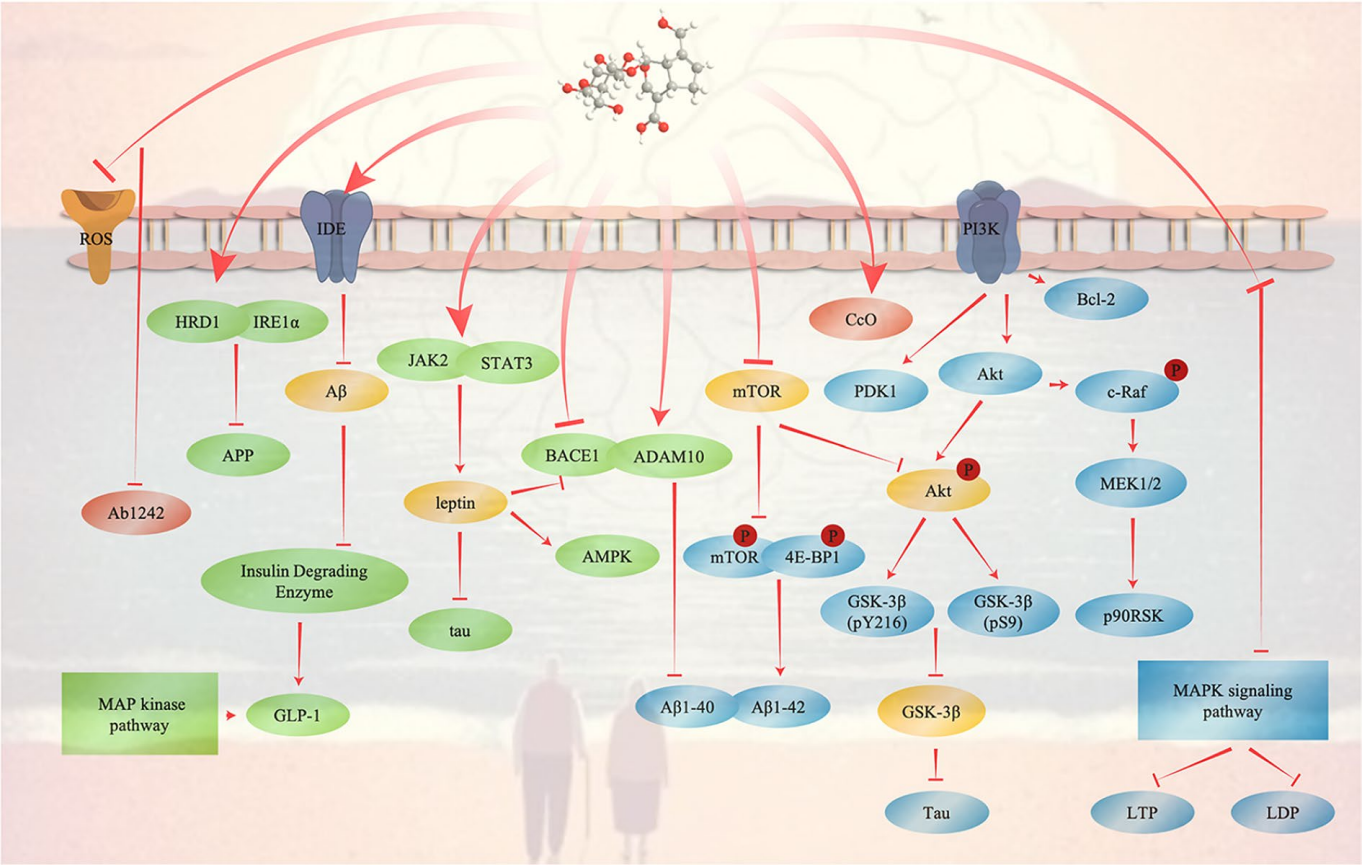
The therapeutic effect of geniposide on Alzheimer's disease. Geniposide increases the expression level of p-4E-BP1 and reduces the expression of p-mTOR and p-Akt to improve cognitive impairment. It can treat AD by directly upregulating the phosphorylation level of Akt and downregulating the phosphorylation level of tau protein

GLP-1 is a brain-gut peptide secreted by ileal endocrine cells. It can bind to G protein-coupled receptors and become a key physiological promoting factor for insulin release [79, 80]. It can not only stimulate the secretion of insulin by pancreatic β cells and inhibit the secretion of glucagon from α cells but also inhibit gastric emptying and food intake. Geniposide controls weight gain and diet-related blood glucose metabolism through the above effects [81, 82]. At present, GLP-1R is considered to be an important pharmacological target for the treatment of type 2 diabetes (T2DM) [83]. Studies have found that the activation of GLP-1R has antidepressant effects and improves cognitive deficits [84]. Therefore, many researchers believe that the development of drugs for the treatment of T2DM may help improve the pathological conditions of AD. Studies have found that geniposide, as a glucagon-like peptide-1 receptor agonist, has a significant effect on improving the pathological process of AD. Geniposide can inhibit tau phosphorylation and oxidative stress by reducing amyloid plaques, reducing memory impairment and synaptic loss, and can also promote neurite growth. In addition, geniposide can exert neuroprotective effects by inhibiting inflammation. The above effect prevents the progression of AD through the GLP-1R signalling pathway [85]. In a previous study, we reported that geniposide had a protective effect on PC12 cells in a cerebral ischaemia model. However, several studies also reported that geniposide could also treat AD by playing a neuroprotective role in PC12 cells. Studies have suggested that oxidative stress plays a key role in the degeneration of neurons in AD, and geniposide plays a neurotrophic and neuroprotective role in oxidative damage induced by H(2)O(2)-induced PC12 cells by increasing the phosphorylation of Akt308, Akt473, GSK-3Beta and PDK1. In addition, geniposide can also inhibit H(2)O(2)-induced PC12 cell apoptosis by inducing the expression of the antiapoptotic protein Bcl-2 [86]. In previous studies, it was also found that geniposide can induce neuronal differentiation in PC12 cells. Their results showed that geniposide not only upregulated the expression of the antiapoptotic proteins Bcl-2 and haem oxygenase-1 (HO-1) but also antagonized the oxidative damage of PC12 cells induced by hydrogen peroxide. The data indicate that geniposide activates the p90RSK transcription factor to regulate the expression of HO-1, Bcl-2 and other antioxidant proteins. Geniposide exerted the above effects through the MAPK signalling pathway to treat AD [87] (Fig. 3).

## Depression

### Main research models

At present, due to the numerous pathogenic factors and complex treatment mechanisms of depression, various models have been established to explore the mechanism of action of geniposide in the treatment of depression. After reviewing many studies, it was found that depression models are mainly divided into two categories: behavioural models and drug-based models. The behavioural models are mainly based on the fact that animals show decreased activity ability after being subjected to severe stress for a long time. Among them, chronic unpredictable mild stress (CUMS) is currently the most direct and effective behavioural depression model. In this model, the variability and unpredictability of stress factors are the key to the model, but the actual operation workload of this model is relatively large, and the duration of the experiment is relatively long [88–90]. Other methods of drug modelling are conventional methods such as gastric gavage and intraperitoneal injection, which are simple in operation and less time-consuming.

### The potential mechanism of geniposide in behavioural depression models

There are studies showing that repeated restraint stress (RRS) is used to induce depression in mice. Studies have shown that geniposide can significantly improve some depression-like behaviours caused by RRS, including the reduction of sucrose preference (SP) in the sucrose preference test (SPT), the rest time of the tail suspension test (TST) and the forced swimming test (FST) is prolonged, and exercise activity in the open field test (OFT) is reduced. In addition, geniposide can inhibit neuronal apoptosis in the hippocampus of mice induced by RRS and downregulate IL-1, cytokines and TNF-α (cell factor) and the expression level of proinflammatory cytokines. Geniposide can upregulate the expression of GLP-1R/protein kinase B (AKT) signalling-related proteins. However, these effects of geniposide can be blocked by the GLP-1R antagonist exendin(9-39) (Ex9-39). These data indicate that the antidepressant effect of geniposide may be closely related to the GLP-1R/AKT signalling pathway [91]. There was also a study on a rat model of depression induced by CUMS for 3 consecutive weeks. The study results showed that 25, 50, and 100 mg/kg geniposide treatments could improve depression-like behaviours caused by CUMS, including improving OFT hybridization and eating behaviours, increasing sugar intake, shortening FST rest time, and prolonging swimming time. In addition, geniposide could improve the hyperactivity of the hypothalamic–pituitary–adrenal (HPA) axis caused by CUMS by downregulating the expression of cortical serum markers, the adrenal index and hypothalamic CRH mRNA, and upregulating the level of hypothalamic GRα mRNA and the expression of GRα protein in the PVN. Based on the above data, geniposide may have an antidepressant therapeutic effect through its effect on the HPA axis [92].

### The potential mechanisms of geniposide in drug-induced depression models

Another study induced antidepressant behaviour in diabetic mice by the administration of streptozotocin. Stz-induced depressive behaviour in diabetic mice was treated by a gavage of geniposide at 50 and 100 mg/kg per day. The results showed that geniposide could reduce the excessive increase in blood glucose and resting time in mice induced by STZ in the FST and could also further upregulate the mRNA expression of brain-derived neurotrophic factor (BDNF) and pro-myosin-associated kinase B (trkB) in diabetic mice. Nevertheless, geniposide had no effect on the STZ-induced increase in serum corticosterone (CORT) levels in diabetic mice [93]. Another study established a diabetes-related depression model through HDF combined with CORT treatment. Geniposide is one of the natural compounds that activates GLP-1R. Studies have found that geniposide could not only enhance the activity of cAMP-response element-binding protein (CREB) in the hippocampus but also improve cognitive dysfunction and depression/anxiety symptoms. However, the use of 666-15 to block CREB activity will reduce the neuroprotective effects of geniposide and the antidepressant-like behavioural effects [94].

Studies using drug-based modelling methods have also found that geniposide could be used in combination with other drugs to treat depression. A depression model was established by intraperitoneal injection of 1 mg/kg LPS. The results of the study showed that the combined application of fluoxetine, geniposide and eleutheroside B could significantly prolong the resting time in the FST and TST in depressed mice, reduce the level of inflammatory factor IL-1 β in the serum, and significantly downregulate the expression of NF-κB gene and protein in hippocampus. In addition, the combination of geniposide and eleutheroside B significantly downregulated rat serum TNF-α levels and hippocampal IDO mRNA and protein expression. These data indicate that the antidepressant effect of geniposide and eleutheroside B may be to inhibit the activation of NF-κB, downregulate the expression of proinflammatory cytokines, and inhibit the neuroinflammatory response. Geniposide can also inhibit IDO expression (a key enzyme in tryptophan metabolism) from affecting tryptophan metabolism [95].

## Other brain diseases

**Fig. 4:**
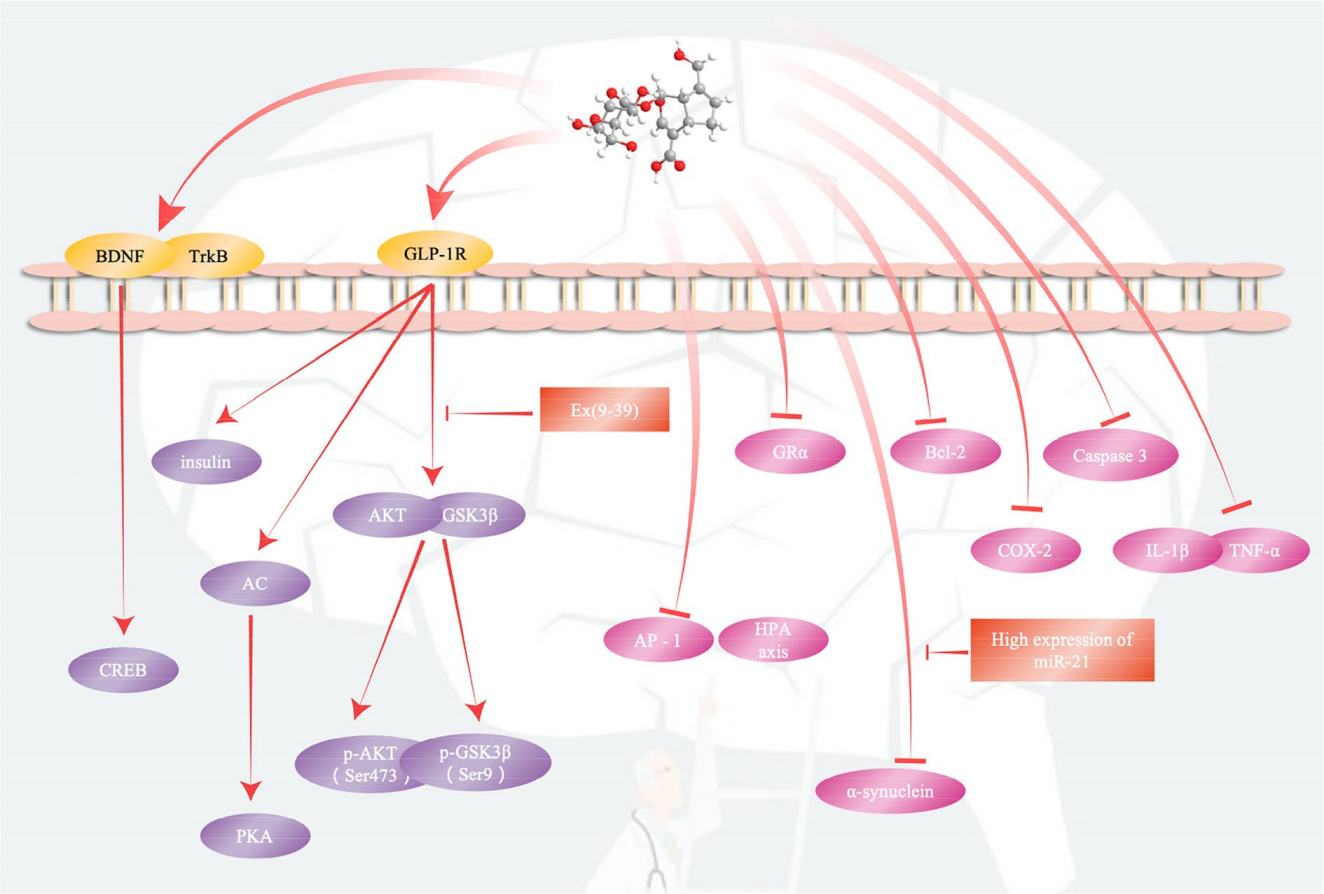
The therapeutic effect of geniposide on depression and other brain diseases. Geniposide downregulates the expression level of proinflammatory cytokines and upregulates the expression of GLP-1R/protein kinase B (AKT) signal-related proteins. It can also further upregulate the mRNA expression of BDNF and TrkB in diabetic mice to treat depressive behaviour

There are also some reports on the therapeutic effects of geniposide on other brain diseases, but the number of reports is relatively small. Therefore, we summarize these studies together for elaboration. Related literature reports that geniposide can improve the pathology of epileptic mice through the PI3K/Akt/GSK-3β signalling pathway. They first induced a mouse epilepsy model with a maximum current (50 mA, 50 Hz, 1 s) and then administered geniposide at doses of 0, 5, 10, and 20 mg/kg to the mice by gavage. The results showed that geniposide could significantly reduce the incidence of clonic seizures in epileptic mice and significantly increase the incubation period of clonic seizures in epileptic mice. In addition, geniposide significantly downregulated the expression of cyclooxygenase-2 mRNA and activator protein 1 in epileptic mice. Geniposide treatment could enhance the activation of Akt and upregulate the protein expression of GSK-3β and PI3K [96]. There are also studies showing that geniposide has a therapeutic effect on mice with Parkinson's disease. Researchers used a 30 mg/kg intraperitoneal injection of 1-methyl-4-phenyl-1,2,3,6-tetrahydropyridine (MPTP) to induce acute Parkinson's disease (PD) in model mice. The results showed that treatment with 100 mg/kg geniposide could improve the slow motion ability and exercise balance ability of mice. Geniposide could also restore the number of tyrosine hydroxylase (TH)-positive dopaminergic neurons in the substantia nigra compact area, upregulate the level of the growth factor signalling molecule Bax and downregulate the expression of the apoptosis signalling molecule Bcl-2 to reduce cell apoptosis, thereby playing a role in the treatment of Parkinson's disease [97]. In addition, there are studies that used similar modelling methods to find that geniposide could increase the expression of lysosomal-associated membrane protein 2 (LAMP2A) protein and mRNA in PD models and reduce the level of α-synuclein protein. Both the cell and mouse models of this study found that geniposide significantly reduced the expression level of miR-21. Geniposide could also inhibit the expression of α-synuclein in the PD model through the miR-21/LAMP2A axis to exert neuroprotective effects [98] (Fig. 4).

## Discussion and outlook

*Gardenia jasminoides* Ellis is the dried and mature fruit of the Rubiaceae plant Gardenia. According to traditional Chinese medicine, it has the function of purging fire and eliminating troubles, clearing heat and dampness, cooling blood and detoxification, and reducing swelling and pain [3, 12, 99]. Geniposide, which is the main component of *Gardenia jasminoides* Ellis, has also received specific attention [12, 100, 101]. It has been proven to have therapeutic potential for various diseases, including liver disease, kidney disease, cardiovascular disease, neurodegenerative diseases, tumours and various types of inflammation throughout the body [13, 102–104]. Combined with modern pharmacological research, the anti-inflammatory, anti-apoptotic, and antioxidant functions of geniposide are very significant and have been reported in many studies [105, 106]. Although the therapeutic prospects of geniposide are very broad, with in-depth research in recent years, geniposide has been found to be hepatotoxic when used in large doses [10, 107, 108]. A study administered 25, 50, or 100 mg/kg geniposide to Sprague–Dawley (SD) rats once daily for 26 weeks. Physiological changes were observed in the 50 and 100 mg/kg geniposide groups starting from week 13. At the end of 26 weeks, geniposide was found to affect serum biochemical indices and urinary analysis and haematological parameters, as well as relative organ weight. More importantly, continuous gavage with 100 mg/kg geniposide for 26 weeks resulted in significant liver and kidney damage [109]. These data show that large doses of geniposide can cause uncontrollable pathological damage. Most studies report that the effectiveness of geniposide is dose-dependent, and high-dose geniposide has a good therapeutic effect. Although there are potential adverse reactions with long-term application of geniposide in large doses, a review of the literature shows that brain protection is apparent in the dose range of 50–100 mg/kg. Therefore, this provides data support for the future use of geniposide, especially in terms of dosage.

In recent years, the combination of geniposide and other drugs has gradually become a research hotspot. The alleviation of potential hepatic toxicity might be one of the main reasons for the combined application. In particular, there are many agents suitable for hepatoprotection in current research [110–112]. On the other hand, the synergistic interaction for stronger efficacy might be the most important issue for combined application with other natural products. For example, we previously reported that the combination of geniposide and berberine, baicalin and other monomers has the potential to enhance the efficacy. The combined use of deoxycholic acid has a stronger therapeutic effect on damaged cells. Geniposide can also be combined with ginsenoside Rg1 to play a protective effect on focal cerebral ischaemia in rats by inhibiting miR-155-5p in microglia after ischaemia injury [110]. Geniposide is often compatible with the above monomers to treat encephalopathy, and its mechanism is network regulation. Using a safe dose of geniposide as an adjuvant, combined with existing therapeutic drugs or traditional Chinese medicine to treat corresponding diseases may be a way to fully access the efficacy and safety of geniposide.

In recent years, the severe effects of brain diseases, such as high mortality and high disability rate, have attracted attention from all over the world, and the neuroprotective effect of geniposide has also been recognized by extensive research. By summarizing various studies, we found that geniposide has a multitarget therapeutic effect on cerebral ischaemia, AD, and depression. Geniposide can reduce the expression of iNOS and NF-κB and inhibit the release of the inflammatory factors TNF-α and IL-6 to exert an anti-inflammatory effect. In a microglial OGD model, we found that geniposide could activate the autophagy activity of microglia by inhibiting the nuclear transcription activity triggered by NF-κB p65, and it could inhibit the expression of inflammatory cytokines to reduce inflammation [42, 43]. Second, geniposide can activate the PI3K/AKT and Wnt/catenin pathways by upregulating the expression of H19 in PC-12 cells in the OGD model, thereby reducing cell damage. Finally, geniposide can improve the expression of related proteins after HI injury by activating the PI3K/Akt signalling pathway, thereby exerting a protective effect on the brain [58].

In the AD model, we found that geniposide enhances autophagy and lysosome clearance of Aβ fibres by downregulating mTOR signalling and can also increase the expression level of p-4E-BP1 and reduce the levels of p-mTOR and p-Akt. The enhancement of these expression levels can improve the exploration and memory abilities of mice and effectively improve cognitive dysfunction. Geniposide can significantly reduce the phosphorylation level of tau protein in the brains of APP/PS1 transgenic mice and improve oxidative stress and mitochondrial dysfunction in mice to prevent the development of AD. In vitro studies have found that geniposide can treat AD by directly upregulating the phosphorylation level of Akt, downregulating the phosphorylation level of tau protein, and enhancing insulin signalling [69, 77].

Similarly, geniposide can significantly improve some depression-like behaviours induced by RRS and inhibit RRS-induced neuronal apoptosis in the hippocampus of mice, downregulate the expression level of proinflammatory cytokines, and upregulate GLP-1R/protein kinase B (AKT) signal-related protein expression. It is also possible to further upregulate the mRNA expression of BDNF and TrkB in diabetic mice to treat depressive behaviour [91, 93].

**Fig. 5:**
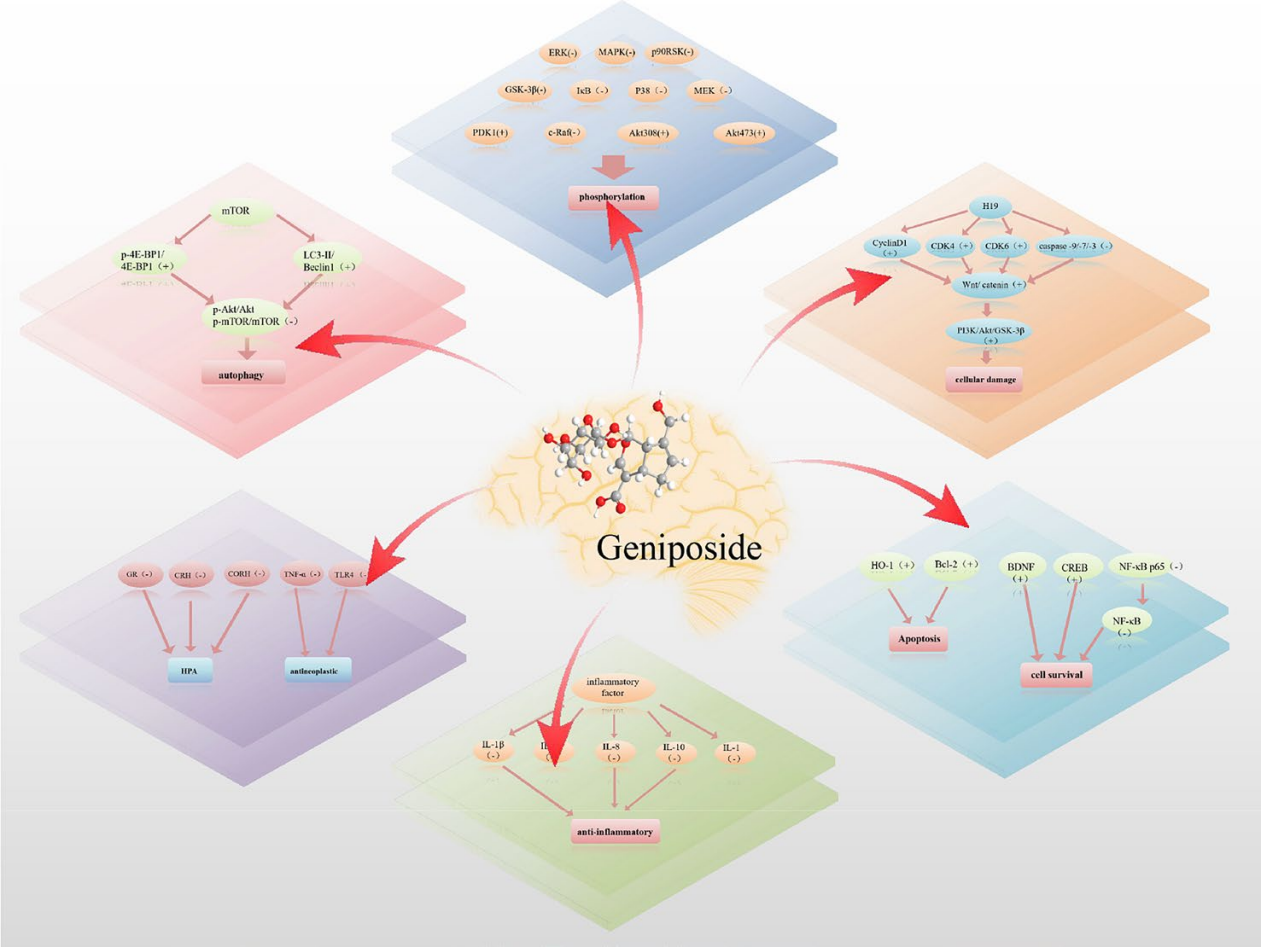
Multitarget function of geniposide in cerebral diseases. Geniposide treats cerebral diseases by exerting antiapoptotic and anti-inflammatory effects, inhibiting the phosphorylation of related factors, and inhibiting cell damage

In summary, geniposide has a multitarget therapeutic effect on cerebral diseases, and its mechanisms and signalling pathways are complicated. In addition, geniposide has great application prospects in inhibiting the expression of inflammatory cytokines and improving oxidative stress and mitochondrial damage (Fig. 5).

## Conclusion

Geniposide has a multi-target therapeutic effect on cerebral diseases, and its therapeutic mechanisms and signal pathways are diverse. Detailed explorations have shown that the biological activities of inhibiting inflammatory cytokine secretion, alleviating oxidative stress, and suppressing mitochondrial damage are also involved in the mechanism of action of geniposide. Therefore, geniposide is a promising agent awaiting further exploration for the treatment of cerebral diseases via various phenotypes or signalling pathways.

## Data Availability

Not available.

## Abbreviations

AD: Alzheimer’s disease
Aβ1-40: Amyloid-β 1-40
AChE: Acetyl-cholinesterase
BBB: Blood Brain Barrier
BDNF: Brain-derived neurotrophic factor
BMECs: Brain microvascular endothelial cells
CcO: Cytochrome c oxidase
ChAT: Choline acetyltransferase
CREB: CAMP-response element binding protein
CUMS: Chronic unpredictable mild stress
ER: Endoplasmic reticulum
ERK1/2: Xtracellular signal-regulated kinase ½
FST: Forced swimming test
GLP-1: Glucagon-like peptide-1
GSK3: Glycogen synthase kinase-3
GLP-1R: Glucagon-like peptide-1 receptor
HI: Hypoxic-ischemic
HO-1: Heme oxygenase-1
HPA: Hypothalamic–pituitary–adrenal
IDE: Insulin degrading enzyme
IL-1: Interleukin-1
IL-1β: Interleukin-1β
IL-6: Interleukin-6
IL-8: Interleukin-8
IL-10: Interleukin-10
iNOS: Inducible nitric oxide synthase
I/R: Ischemia/reperfusion
LA: Leptin antagonist
LAMP2A: Lysosomal-associated membrane protein 2
LPS: Lipopolysaccharide
MCAO: Middle cerebral artery occlusion
MCA: Middle cerebral artery
MCP-1: Monocyte chemotactic protein 1
MEK1/2: Mitogen activated protein kinase ½
MMP: Mitochondrial membrane potential
MMP-9: Matrix metallopeptidase-9
MPTP: 1-Methyl-4-phenyl-1,2,3,6-tetrahydropyridine
mTOR: Mammalian target of rapamycin
NF-κB: Nuclear factor-kappa B
NLRP3: Nod-like receptor protein 3
NMDAR: *N*-methyl-d-aspartate receptor
OFT: Open air test
OGD: Oxygen-glucose-deprivation
PD: Parkinson’s disease
RRS: Repeated restraint stress
pMCAO: Permanent middle cerebral artery occlusion
SD: Sprague–Dawley
SP: Sucrose preference
SPT: Sucrose preference test
STZ: Streptozotocin
T2DM: Type 2 diabetes
TH: Tyrosine hydroxylase
TLR4: Toll-like receptor 4
tMCAO: Transient middle cerebral artery occlusion
TNF-α: Tumor necrosis factor-α
tPA: Tissue plasminogen activator
TST: Tail suspension test
TUDCA: Tauroursodeoxycholic acid
UPR: Unfolded protein response

## References

[1] Ziemska J, Szynal T, Mazańska M, Solecka J (2019). Natural medicinal resources and their therapeutic applications. Rocz Panstw Zakl Hig..

[2] Wang W, Liang YS (2016). Artemisinin: a wonder drug from Chinese natural medicines. Chin J Nat Med..

[3] Chen L, Li M, Yang Z, Tao W, Wang P, Tian X (2020). Gardenia jasminoides Ellis: Ethnopharmacology, phytochemistry, and pharmacological and industrial applications of an important traditional Chinese medicine. J Ethnopharmacol..

[4] Cui Y, Wang Q, Wang M, Jia J, Wu R (2019). Gardenia decoction prevent intestinal mucosal injury by inhibiting pro-inflammatory cytokines and NF-κB signaling. Front Pharmacol..

[5] Chen S, Sun P, Zhao X, Yi R, Qian J, Shi Y (2017). Gardenia jasminoides has therapeutic effects on L-NNA-induced hypertension in vivo. Mol Med Rep.

[6] Xiao W, Li S, Wang S, Ho CT (2017). Chemistry and bioactivity of *Gardenia jasminoides*. J Food Drug Anal.

[7] Li KD, Wang QS, Zhang WW, Zhang WY, Fu SN, Xu D (2020). Gardenia fructus antidepressant formula for depression in diabetes patients: a systematic review and meta-analysis. Complement Ther Med..

[8] Kim HI, Hong SH, Ku JM, Kim MJ, Ju SW, Chang SW (2020). *Gardenia jasminoides* enhances CDDP-induced apoptosis of glioblastoma cells via AKT/mTOR pathway while protecting death of astrocytes. Nutrients.

[9] Habtemariam S, Lentini G (2018). Plant-derived anticancer agents: Lessons from the pharmacology of geniposide and its aglycone, genipin. Biomedicines..

[10] Shan M, Yu S, Yan H, Guo S, Xiao W, Wang Z (2017). A review on the phytochemistry, pharmacology, pharmacokinetics and toxicology of geniposide, a natural product. Molecules.

[11] Hasso AN, Stringer WA, Brown KD. Cerebral ischemia and infarction. Neuroimaging Clin; 1994. 733–52. 7858918

[12] Li N, Li L, Wu H, Zhou H (2019). Antioxidative property and molecular mechanisms underlying geniposide-mediated therapeutic effects in diabetes mellitus and cardiovascular disease. Oxid Med Cell Longev..

[13] Chen Z, Liu W, Qin Z, Liang X, Tian G (2020). Geniposide exhibits anticancer activity to medulloblastoma cells by downregulating microRNA-373. J Biochem Mol Toxicol.

[14] Lubrini G, Martín-Montes A, Díez-Ascaso O, Díez-Tejedor E (2018). Brain disease, connectivity, plasticity and cognitive therapy: a neurological view of mental disorders. Neurologia SEGO.

[15] March PA (1996). Degenerative brain disease. Vet Clin North Am Small Anim Pract..

[16] Kovacic JC, Castellano JM, Fuster V (2012). The links between complex coronary disease, cerebrovascular disease, and degenerative brain disease. Ann N Y Acad Sci.

[17] Wells CE (1978). Chronic brain disease: an overview. Am J Psychiatry.

[18] Sveinsson OA, Kjartansson O, Valdimarsson EM (2014). Cerebral ischemia/infarction-diagnosis and treatment. Laeknabladid..

[19] Sveinsson OA, Kjartansson O, Valdimarsson EM (2014). Cerebral ischemia/infarction-epidemiology, causes and symptoms. Laeknabladid.

[20] Wang M, Liu JX, Yao MJ, Ren JG (2020). Advances in research on pharmacological and neuroprotective effects of traditional Chinese medicine after cerebral ischemia. Zhongguo Zhongyao Zazhi. Zhongguo Zhongyi Yanjiuyuan.

[21] Hasso AN, Stringer WA, Brown KD (1994). Cerebral ischemia and infarction. Neuroimaging Clin..

[22] Lyden PD, Zivin JA (1993). Hemorrhagic transformation after cerebral ischemia: mechanisms and incidence. Cerebrovasc Brain Metab Rev..

[23] Li J, Xu J, Liu Z, Zou Z, Jin M, Tao T (2020). HIF-1α attenuates neuronal apoptosis by upregulating EPO expression following cerebral ischemia-reperfusion injury in a rat MCAO model. Int J Mol Med..

[24] Jiang Y, Wen J, Zhang W, Ma Z, Zhang C, Wang J (2020). Metabolomics coupled with integrative pharmacology reveals the therapeutic effect of l-borneolum against cerebral ischaemia in rats. J Pharm Pharmacol..

[25] Bondi MW, Edmonds EC, Salmon DP (2017). Alzheimer’s disease: past, present, and future. J Int Neuropsychol Soc..

[26] Harrìson C, Charles J, Britt H (2008). AD(H)D. Aust Fam Physician..

[27] Sarris J, O’Neil A, Coulson CE, Schweitzer I, Berk M (2014). Lifestyle medicine for depression. BMC Psychiatry..

[28] Raič M (2017). Depression and heart diseases: leading health problems. Psychiatr Danub..

[29] Zhang Y, Chen Y, Ma L (2018). Depression and cardiovascular disease in elderly: current understanding. J Clin Neurosci.

[30] Lopez MS, Vemuganti R (2018). Modeling transient focal ischemic stroke in rodents by intraluminal filament method of middle cerebral artery occlusion. Methods Mol Biol.

[31] Xiang C, Zhang Y, Guo W, Liang XJ (2020). Biomimetic carbon nanotubes for neurological disease therapeutics as inherent medication. Acta Pharm Sin B..

[32] McCullough LD, Liu F (2011). Middle cerebral artery occlusion model in rodents: methods and potential pitfalls. J Biomed Biotechnol..

[33] Sun K, Fan J, Han J (2015). Ameliorating effects of traditional Chinese medicine preparation, Chinese materia medica and active compounds on ischemia/reperfusion-induced cerebral microcirculatory disturbances and neuron damage. Acta Pharm Sin B.

[34] Fluri F, Schuhmann MK, Kleinschnitz C (2015). Animal models of ischemic stroke and their application in clinical research. Drug Des Devel Ther.

[35] Ma R, Xie Q, Li Y, Chen Z, Ren M, Chen H (2020). Animal models of cerebral ischemia: a review. Biomed Pharmacother..

[36] Kalogeris T, Baines CP, Krenz M, Korthuis RJ (2017). Ischemia/reperfusion. Compr Physiol.

[37] Galkin A (2019). Brain Ischemia/reperfusion injury and mitochondrial complex I damage. Biochem.

[38] Ryou M, Mallet RT (2018). An in vitro oxygen–glucose deprivation model for studying ischemia–reperfusion injury of neuronal cells. Methods Mol Biol..

[39] Tabakman R, Jiang H, Shahar I, Arien-Zakay H, Levine RA, Lazarovici P (2005). Neuroprotection by NGF in the PC12 in vitro OGD model: Involvement of mitogen-activated protein kinases and gene expression. Ann N Y Acad Sci..

[40] Salvador E, Burek M, Förster CY (2018). An In Vitro Model of Traumatic Brain Injury. Galdos Meow..

[41] Huang B, Chen P, Huang L, Li S, Zhu R, Sheng T (2017). Geniposide attenuates post-ischaemic neurovascular damage via GluN2A/AKT/ ERK-dependent mechanism. Cell Physiol Biochem.

[42] Li LJ, Han ZF, Li LX, Yan B (2020). Effects of geniposide on the neuroinflammation in chronic cerebral hypoperfusion rat model. J Sichuan Univ..

[43] Wang J, Hou J, Zhang P, Li D, Zhang C, Liu J (2012). Geniposide reduces inflammatory responses of oxygen-glucose deprived rat microglial cells via inhibition of the TLR4 signaling pathway. Neurochem Res.

[44] Wu W, He X, Xie S, Li B, Chen J, Qu Y (2020). Protective effects of Huang-Lian-Jie-Du-Tang against Aβ25–35-induced memory deficits and oxidative stress in rats. J Int Med Res..

[45] Lee IJ, Chao CY, Yang YC, Cheng JJ, Huang CL, Chiou CT (2021). Huang Lian Jie Du Tang attenuates paraquat-induced mitophagy in human SH-SY5Y cells: a traditional decoction with a novel therapeutic potential in treating Parkinson’s disease. Biomed Pharmacother..

[46] Pan L, Zhou J, Zhu H, Wang W, Zhang M, Tian X (2014). Study on integrated pharmacokinetics of gardenia acid and geniposide: time-antioxidant efficacy after oral administration of huanglian-zhizi couplet medicine from huang-lian-jie-du-tang in MCAO rats. Am J Chin Med.

[47] Pan L, Wang W, Shi F, Zhou J, Zhang M, Zhu H, et al. Exploratory pharmacokinetics of geniposide in rat model of cerebral ischemia orally administered with or without baicalin and/or berberine. Evid Based Complement Altern Med. 2013;2013. 10.1155/2013/349531 PMC3866786 24367386

[48] Zhang Q, Fu X, Wang J, Yang M, Kong L (2017). Treatment effects of ischemic stroke by berberine, baicalin, and jasminoidin from Huang-Lian-Jie-Du-Decoction (HLJDD) explored by an integrated metabolomics approach. Oxid Med Cell Longev..

[49] Li C, Wang X, Cheng F, Du X, Yan J, Zhai C (2019). Geniposide protects against hypoxia/reperfusion-induced blood-brain barrier impairment by increasing tight junction protein expression and decreasing inflammation, oxidative stress, and apoptosis in an in vitro system. Eur J Pharmacol..

[50] Luo X, Wu S, Jiang Y, Wang L, Li G, Qing Y (2020). Inhibition of autophagy by geniposide protects against myocardial ischemia/reperfusion injury. Int Immunopharmacol..

[51] Prinz M, Jung S, Priller J (2019). Microglia biology: one century of evolving concepts. Cell..

[52] Wolf SA, Boddeke HWGM, Kettenmann H (2017). Microglia in physiology and disease. Annu Rev Physiol.

[53] Nayak D, Roth TL, McGavern DB (2014). Microglia development and function. Annu Rev Immunol.

[54] Fu C, Zhang X, Lu Y, Wang F, Xu Z, Liu S (2020). Geniposide inhibits NLRP3 inflammasome activation via autophagy in BV-2 microglial cells exposed to oxygen–glucose deprivation/reoxygenation. Int Immunopharmacol..

[55] Shen X, Zhao YF, Xu SQ, Wang L, Cao HM, Cao Y (2019). Cathepsin L induced PC-12 cell apoptosis via activation of B-Myb and regulation of cell cycle proteins. Acta Pharmacol Sin..

[56] Jung J, Jeongbin SEO, Joungmok KIM, Kim JHEE (2018). Ursolic acid causes cell death in PC-12 cells by inducing apoptosis and impairing autophagy. Anticancer Res.

[57] Li R, Yin F, Guo Y, Ruan Q, Zhu Q (2018). Angelica polysaccharide protects PC-12 cells from lipopolysaccharide-induced injury via down-regulating microRNA-223. Biomed Pharmacother.

[58] Yuan Y, Zheng Z (2019). Geniposide protects PC-12 cells against oxygen and glucose deprivation-induced injury by up-regulation of long-noncoding RNA H19. Life Sci.

[59] Li F, Li W, Li X, Li F, Zhang L, Wang B (2016). Geniposide attenuates inflammatory response by suppressing P2Y14 receptor and downstream ERK1/2 signaling pathway in oxygen and glucose deprivation-induced brain microvascular endothelial cells. J Ethnopharmacol.

[60] Wu J, Wang B, Li M, Shi YH, Wang C, Kang YG (2019). Network pharmacology identification of mechanisms of cerebral ischemia injury amelioration by Baicalin and Geniposide. Eur J Pharmacol..

[61] Cheng F, Ma C, Sun L, Zhang X, Zhai C, Li C (2018). Synergistic neuroprotective effects of geniposide and ursodeoxycholic acid in hypoxia-reoxygenation injury in SH-SY5Y cells. Exp Ther Med.

[62] Chen L, Xu S, Wu T, Shao Y, Luo L, Zhou L (2019). Studies on APP metabolism related to age-associated mitochondrial dysfunction in APP/PS1 transgenic mice. Aging.

[63] Xiang J, Cao K, Dong YT, Xu Y, Li Y, Song H (2020). Lithium chloride reduced the level of oxidative stress in brains and serums of APP/PS1 double transgenic mice via the regulation of GSK3β/Nrf2/HO-1 pathway. Int J Neurosci.

[64] Oakley H, Cole SL, Logan S, Maus E, Shao P, Craft J (2006). Intraneuronal β-amyloid aggregates, neurodegeneration, and neuron loss in transgenic mice with five familial Alzheimer’s disease mutations: potential factors in amyloid plaque formation. J Neurosci.

[65] Lenzen S (2008). The mechanisms of alloxan- and streptozotocin-induced diabetes. Diabetologia.

[66] Grieb P (2016). Intracerebroventricular streptozotocin injections as a model of Alzheimer’s disease: in search of a relevant mechanism. Neurobiol..

[67] Baig MA, Panchal SS (2019). Streptozotocin-induced diabetes mellitus in neonatal rats: an insight into its applications to induce diabetic complications. Curr Diabetes Rev.

[68] Zhang Z, Wang X, Zhang D, Liu Y, Li L (2019). Geniposide-mediated protection against amyloid deposition and behavioral impairment correlates with downregulation of mTOR signaling and enhanced autophagy in a mouse model of Alzheimer’s disease. Aging.

[69] Zhang Z, Gao W, Wang X, Zhang D, Liu YZ, Li L (2020). Geniposide effectively reverses cognitive impairment and inhibits pathological cerebral damage by regulating the mTOR Signal pathway in APP∕PS1 mice. Neurosci Lett..

[70] Zhang Y, Yin F, Liu J, Liu Z (2016). Geniposide attenuates the phosphorylation of tau protein in cellular and insulin-deficient APP/PS1 transgenic mouse model of Alzheimer’s disease. Chem Biol Drug Des.

[71] Zhao C, Zhang H, Li H, Lv C, Liu X, Li Z (2017). Geniposide ameliorates cognitive deficits by attenuating the cholinergic defect and amyloidosis in middle-aged Alzheimer model mice. Neuropharmacology.

[72] Lv C, Liu X, Liu H, Chen T, Zhang W (2014). Geniposide attenuates mitochondrial dysfunction and memory deficits in APP/PS1 transgenic mice. Curr Alzheimer Res..

[73] Zhang Y, Yin F, Liu J, Liu Z, Guo L, Xia Z (2015). Geniposide attenuates insulin-deficiency-induced acceleration of β-amyloidosis in an APP/PS1 transgenic model of Alzheimer’s disease. Neurochem Int.

[74] Gao C, Liu Y, Jiang Y, Ding J, Li L (2014). Geniposide ameliorates learning memory deficits, reduces tau phosphorylation and decreases apoptosis via GSK3β Pathway in streptozotocin-induced alzheimer rat model. Brain Pathol.

[75] Liu J, Zhang Y, Deng X, Yin F (2013). Geniposide decreases the level of Aβ1-42 in the hippocampus of streptozotocin-induced diabetic rats. Acta Biochim Biophys Sin.

[76] Cui H, Deng M, Zhang Y, Yin F, Liu J (2018). Geniposide increases unfolded protein response-mediating HRD1 expression to accelerate APP degradation in primary cortical neurons. Neurochem Res..

[77] Liu J, Liu Z, Zhang Y, Yin F (2015). Leptin signaling plays a critical role in the geniposide-induced decrease of tau phosphorylation. Acta Biochim Biophys Sin.

[78] Liu Z, Zhang Y, Liu J, Yin F (2017). Geniposide attenuates the level of Aβ1–42 via enhancing leptin signaling in cellular and APP/PS1 transgenic mice. Arch Pharm Res..

[79] Arcones AC, Vila-Bedmar R, Mirasierra M, Cruces-Sande M, Vallejo M, Jones B (2021). GRK2 regulates GLP-1R-mediated early phase insulin secretion in vivo. BMC Biol BMC Biol.

[80] Willard FS, Ho JD, Sloop KW (2020). Discovery and pharmacology of the covalent GLP-1 receptor (GLP-1R) allosteric modulator BETP: a novel tool to probe GLP-1R pharmacology. Adv Pharmacol..

[81] Drucker DJ (2018). Mechanisms of action and therapeutic application of glucagon-like peptide-1. Cell Metab..

[82] Doggrell SA (2018). Do glucagon-like peptide-1 receptor (GLP-1R) agonists have potential as adjuncts in the treatment of type 1 diabetes?. Expert Opin. Pharmacother..

[83] Jones B, Buenaventura T, Kanda N, Chabosseau P, Owen BM, Scott R (2018). Targeting GLP-1 receptor trafficking to improve agonist efficacy. Nat Commun..

[84] Trammell TS, Henderson NL, Madkour HS, Stanwood GD, Graham DL (2020). GLP-1R activation alters performance in cognitive tasks in a sex-dependent manner. Neurol Sci..

[85] Liu W, Li G, Hölscher C, Li L (2015). Neuroprotective effects of geniposide on Alzheimer’s disease pathology. Rev Neurosci.

[86] Liu JH, Yin F, Guo LX, Deng XH, Hu YH (2009). Neuroprotection of geniposide against hydrogen peroxide induced PC12 cells injury: involvement of PI3 kinase signal pathway. Acta Pharmacol Sin.

[87] Liu J, Yin F, Zheng X, Jing J, Hu Y (2007). Geniposide, a novel agonist for GLP-1 receptor, prevents PC12 cells from oxidative damage via MAP kinase pathway. Neurochem Int.

[88] Antoniuk S, Bijata M, Ponimaskin E, Wlodarczyk J (2019). Chronic unpredictable mild stress for modeling depression in rodents: meta-analysis of model reliability. Neurosci Biobehav Rev Elsevier Ltd.

[89] Colodro-Conde L, Couvy-Duchesne B, Zhu G, Coventry WL, Byrne EM, Gordon S (2018). A direct test of the diathesis–stress model for depression. Mol Psychiatry.

[90] Pitsillou E, Bresnehan SM, Kagarakis EA, Wijoyo SJ, Liang J, Hung A (2020). The cellular and molecular basis of major depressive disorder: towards a unified model for understanding clinical depression. Mol Biol Rep..

[91] Zhao Y, Li H, Fang F, Qin T, Xiao W, Wang Z (2018). Geniposide improves repeated restraint stress-induced depression-like behavior in mice by ameliorating neuronal apoptosis via regulating GLP-1R/AKT signaling pathway. Neurosci Lett.

[92] Cai L, Li R, Tang WJ, Meng G, Hu XY, Wu TN (2015). Antidepressant-like effect of geniposide on chronic unpredictable mild stress-induced depressive rats by regulating the hypothalamus-pituitary-adrenal axis. Eur Neuropsychopharmacol.

[93] Wang J, Duan P, Cui Y, Li Q, Shi Y (2016). Geniposide alleviates depression-like behavior via enhancing BDNF expression in hippocampus of streptozotocin-evoked mice. Metab Brain Dis Metabolic Brain Disease.

[94] Sun B, Jia X, Yang F, Ren G, Wu X (2020). CREB-mediated generation and neuronal growth regulates the behavioral improvement of geniposide in diabetes-associated depression mouse model. Neurosci Res..

[95] Zhang B, Chang HS, Hu KL, Yu X, Li LN, Xu XQ (2019). Combination of geniposide and eleutheroside B exerts antidepressant-like effect on lipopolysaccharide-induced depression mice model. Chin J Integr Med..

[96] Wei H, Duan G, He J, Meng Q, Liu Y, Chen W (2018). Geniposide attenuates epilepsy symptoms in a mouse model through the PI3K/Akt/GSK-3β signaling pathway. Exp Ther Med.

[97] Chen Y, Zhang Y, Li L, Hölscher C (2015). Neuroprotective effects of geniposide in the MPTP mouse model of Parkinson’s disease. Eur J Pharmacol.

[98] Su C, Yang X, Lou J (2016). Geniposide reduces α-synuclein by blocking microRNA-21/lysosome-associated membrane protein 2A interaction in Parkinson disease models. Brain Res.

[99] Zhou Y-X, Zhang R-Q, Rahman K, Cao Z-X, Zhang H, Peng C (2019). Diverse pharmacological activities and potential medicinal benefits of geniposide. Evid Based Complement Alternat Med.

[100] He T, Shen H, Zhu J, Zhu Y, He Y, Li Z (2019). Geniposide attenuates cadmium-induced oxidative stress injury via Nrf2 signaling in osteoblasts. Mol Med Rep.

[101] Hu L, Zhao J, Liu Y, Liu X, Lu Q, Zeng Z (2020). Geniposide inhibits proliferation and induces apoptosis of diffuse large B-cell lymphoma cells by inactivating the HCP5/miR-27b-3p/MET axis. Int J Med Sci.

[102] Ma S, Zhang C, Zhang Z, Dai Y, Gu R, Jiang R (2019). Geniposide protects PC12 cells from lipopolysaccharide-evoked inflammatory injury via up-regulation of miR-145–5p. Artif Cells Nanomed Biotechnol..

[103] Zhang C, Wang N, Tan HY, Guo W, Chen F, Zhong Z (2020). Direct inhibition of the TLR4/MyD88 pathway by geniposide suppresses HIF-1α-independent VEGF expression and angiogenesis in hepatocellular carcinoma. Br J Pharmacol.

[104] Li Y, Pan H, Zhang X, Wang H, Liu S, Zhang H (2019). Geniposide improves glucose homeostasis via regulating FoxO1/PDK4 in skeletal muscle. J Agric Food Chem.

[105] Kang MJ, Khanal T, Kim HG, Lee DH, Yeo HK, Lee YS (2012). Role of metabolism by human intestinal microflora in geniposideinduced toxicity in HepG2 cells. Arch Pharm Res..

[106] Tian J, Zhu J, Yi Y, Li C, Zhang Y, Zhao Y (2017). Dose-related liver injury of Geniposide associated with the alteration in bile acid synthesis and transportation. Sci Rep..

[107] Hu YZ, Li DF, Zhang Y, Wei JY, Yang HJ (2019). Marker genes of geniposide-induced hepatotoxicity based on genomic strategy. Zhongguo Zhongyao Zazhi. Zhongguo Zhongyi Yanjiuyuan.

[108] Li Y, Pan H, Li X, Jiang N, Huang L, Lu Y (2019). Role of intestinal microbiota-mediated genipin dialdehyde intermediate formation in geniposide-induced hepatotoxicity in rats. Toxicol Appl Pharmacol..

[109] Tian J, Yi Y, Zhao Y, Li C, Zhang Y, Wang L (2018). Oral chronic toxicity study of geniposide in rats. J Ethnopharmacol.

[110] Ma X, Jiang Y, Wen J, Zhao Y, Zeng J, Guo Y (2020). A comprehensive review of natural products to fight liver fibrosis: alkaloids, terpenoids, glycosides, coumarins and other compounds. Eur J Pharmacol..

[111] Ma X, Zhang W, Jiang Y, Wen J, Wei S, Zhao Y (2020). Article 531 1 (2020) paeoniflorin, a natural product with multiple targets in liver diseases-a mini review. Front Pharmacol.

[112] Ma X, Jiang Y, Zhang W, Wang J, Wang R, Wang L (2020). Natural products for the prevention and treatment of cholestasis: a review. Phyther Res..

